# *Vitis vinifera* (Vine Grape) as a Valuable Cosmetic Raw Material

**DOI:** 10.3390/pharmaceutics15051372

**Published:** 2023-04-29

**Authors:** Marta Sharafan, Magdalena A. Malinowska, Halina Ekiert, Beata Kwaśniak, Elżbieta Sikora, Agnieszka Szopa

**Affiliations:** 1 Department of Organic Chemistry and Technology, Faculty of Chemical Engineering and Technology, Cracow University of Technology, Warszawska 24, 31-155 Kraków, Poland; marta.sharafan@doktorant.pk.edu.pl (M.S.);; 2 Department of Pharmaceutical Botany, Medical College, Jagiellonian University, Medyczna 9, 30-688 Kraków, Poland

**Keywords:** *Vitis vinifera*, vine grape, *V. vinifera*-based natural cosmetics, natural raw materials, cosmetic raw materials, phytochemical composition, biological activity

## Abstract

This review refers to botanical, ecological and phytochemical characteristics of *Vitis vinifera* L. (vine grape)–a species, the valuable properties of which are widely exploited in the food industry and in recent times in medicine as well as in phytocosmetology. The general characteristic of *V. vinifera*, followed by the chemical composition and biological activities of different extracts obtained from the plant (fruit, skin, pomace, seed, leaf and stem extracts), are provided. A con*cis*e review of the extraction conditions of grape metabolites and the methods of their analysis are also presented. The biological activity of *V. vinifera* is determined by the presence of high contents of polyphenols, mainly flavonoids (e.g., quercetin, kaempferol), catechin derivatives, anthocyanins and stilbenoids (e.g., *trans*-resveratrol, *trans-ε*-viniferin). The review pays particular attention to the application of *V. vinifera* in cosmetology. It has been proven that *V. vinifera* possesses strong cosmetological-related properties, such as anti-ageing properties, anti-inflammatory properties and skin-whitening properties. Moreover, a review of studies on *V. vinifera* biological activities, which are of particular interest for dermatologic problems, are disclosed. Furthermore, the work also emphasises the importance of biotechnological studies on *V. vinifera.* The last part of the review is addressed to the safety of the use of *V. vinifera*.

## 1. Introduction

Modern phytocosmetology is an extremely prominent field that has recently drawn the attention of research centres. There is an increasing demand for natural cosmetics, the key task of which is to protect the skin from free radicals and oxidative stress with a low risk of side effects [1,2]. Scientists are constantly searching for innovative raw materials with potential applications in different cosmetic formulations [3]. *Vitis vinifera* L. (vine grape, Vitaceae) is one of the best-known fruit crops with wide applications in the food, pharmaceutical and cosmetic industries. The main cultivation regions of *V. vinifera* are located in Europe (France, Italy and Spain), Asia (China) and the Americas (United States, Argentina and Chile) [4].

*V. vinifera* is a rich source of secondary metabolites, particularly flavonoids (flavan-3-ols, flavonols), phenolic acids, anthocyanins, fatty acids, amino acids and vitamins. It also contains the characteristic stilbene derivatives. In addition, the qualitative differences in the phytochemical content depend on the morphological part of the plant [5]. Due to the presence of the above-mentioned groups of compounds, *V. vinifera* is an object of specific scientific interest. It possesses antioxidant, antibacterial, anti-inflammatory and anticancer activities. Moreover, it exhibits cardioprotective, hepatoprotective and neuroprotective properties [6,7,8,9,10].

*V. vinifera* is listed in no pharmacopeia, although there are monographs published by the EMA–HMPC (European Medicines Agency Committee on Herbal Medical Products), the FDA (Food and Drug Administration), and the EFSA (European Food Safety Authority) [11,12,13].

In the pharmaceutical industry, *V. vinifera* is a source of raw materials used due to antioxidative, cardioprotective, hepatoprotective, anticancer, antibacterial and antiviral activities. There are reports of the potential application of *V. vinifera* or the derived active compounds obtained from the plant as eco-friendly, antibacterial or anticancer agents [14,15].

In the food industry, *V. vinifera* can be used as a nutritional supplement or food colouring additive [16]. Due to the antibacterial activity of *V. vinifera*, it can be proposed as a replacement for chemical preservatives [17]. The fruits of *V. vinifera* (grapes) are also widely used for the production of wines, juices and raisins (dried fruits) [18].

*V. vinifera* is also used as an active ingredient in the cosmetics industry, which is determined mainly by its valuable antioxidant, antibacterial and skin-conditioning properties [16,19]. According to the CosIng (Cosmetic Ingredients) database, there are nine forms of raw materials obtained from *V. vinifera* that can be used in cosmetics. Grape extracts can be used as emollients, humectants, emulsifiers, colour additives or fragrances. For instance, *V. vinifera* seeds are recommended as a colouring agent, humectant or hair- and skin-conditioning agent [20]. Cosmetics based on *V. vinifera* are mild and have no skin-irritating properties. The scientific studies demonstrated antioxidant, skin-whitening, anti-inflammatory and anti-ageing activities of the plant, which mainly come from the rich phenolic and stilbenoid composition [7,21,22,23].

The aim of this work is to emphasise the cosmetic applications of *V. vinifera* because the raw materials derived from this species are increasingly valued in the cosmetic industry.

In this work, the ecological, morphological and phytochemical characteristics of *V. vinifera* are presented. Furthermore, particular attention is placed on the cosmetic properties of *V. vinifera* and applications based on scientific studies. Characterisations of the selected cosmetic formulations containing *V. vinifera* extract are presented. Moreover, the biotechnological potential of *V. vinifera* is evaluated. The final part of the work is focused on the *V. vinifera* safety of use.

## 2. General Characteristics

### 2.1. Botanical Characteristic

*Vitis vinifera* L. (vine grape, Vitaceae) is a climbing vine with shoots reaching ten to forty metres long in the natural environment. The main shoot is thick, woody and covered with brown or grey-brown bark. The shoot branching is multi-axis and often bare. The main shoot is pushed back by the side branches creating the tendrils which help the vine to attach to the support. The shoots can be short or long [24].

The leaves are simple, long-tailed and subalternate on the shoots. The pedicles are bare and 4–8 cm long. There are bracts at the root which fall fast. The length and width of the leaf blades are similar and range from 5–15 cm. The lobes vary in size and often overlap, and the sinuses which separate them are rounded. The leaf tooth is large, bare and glabrous, and the middle lobe has a pointed tip. The leaves are usually dark green and hairy but become bare over time. The underside of the leaf blade is light green and sparsely grey-haired [24,25].

The flowers are arranged into 10–20 cm long panicles. The inflorescences are 10–20 cm long and have spidery or bare 1–5 cm long peduncles. The flowers are small and inconspicuous. The flower calyx is five-sided and small. The corolla consists of five petals that are fused at the top. The petals are yellow–green and reach 1.5 mm in length. The staminal or monoecious flowers have five stamens. The flowers are self-pollinating or insect-pollinated [24].

*V. vinifera* fruits have elliptical or globose shape and are 1.5 to 2 cm long in diameter. The fruits are also variable in size and colour. Cultivated varieties can have fruits of red, pink, purple and light green colour. The fruits contain two to four seeds. The pulp is juicy, sweet or sour [24,25].

The seeds have an oval or pear shape with a pointed tip. They can reach up to 6 mm in length [24,25].

### 2.2. Ecological Characteristic

It is accepted that *V. vinifera* is derived from a wild grapevine–*V. vinifera* spp. *sylvestris.* Wild grapevines still naturally occur in small populations in forest areas near rivers, and they climb trees. They are distributed from the Atlantic coast of Europe to Tajikistan and the Western Himalayas. The demand for grape fruits resulted in the domestication of *V. vinifera* spp. *sylvestris* to obtain fruits of better parameters. Due to the hybridisation of different varieties, nowadays, the determination of the point of origin and the spread of *V. vinifera* is complicated. It is assumed that the domestication of *V. vinifera* spp. *sylvestris* took place in the area between the Black Sea and Iran, where *V. vinifera* may have spread to the Near and Middle East and Central Europe [26,27].

Currently, *V. vinifera* is one of the most popular horticultural and crop plants in the world. There are around 10,000 *V. vinifera* varieties, which are planted in numerous countries and grow on six continents with large-scale cultivation areas in Europe, the Middle East and Asia. The main cultivation regions of *V. vinifera* are Spain, France, Italy, China, the United States, Argentina, Chile, Portugal, Romania, Australia, South Africa, Greece, Germany, Brazil and Hungary. The most popular crop varieties are Kyoho, Cabernet, Sauvignon and Sultana [4,28].

### 2.3. Chemical Characteristic

**Figure 1:**
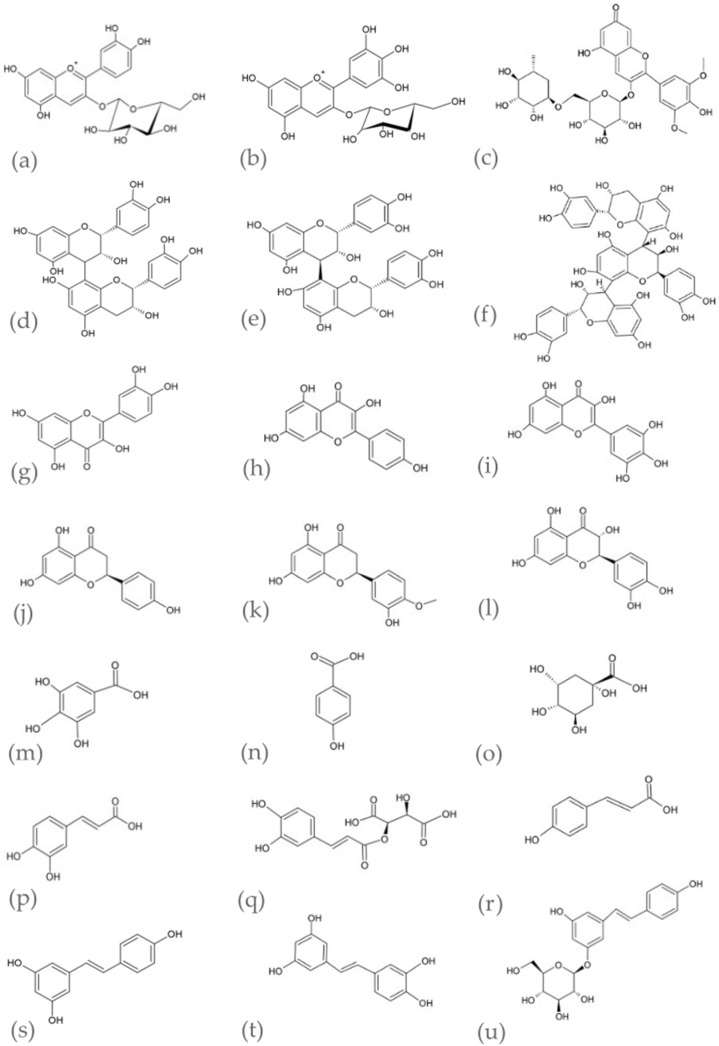
The chemical structure of the selected *V. vinifera* characteristic compounds: anthocyanins–cyanidin 3-*O*-glucoside (**a**), delphinidin 3-*O*-glucoside (**b**), malvidin-3-*O*-rutinoside (**c**); flavan-3-ols-procyanidin B1 (**d**), procyanidin B2 (**e**), procyanidin C (**f**); flavonols-quercetin (**g**), kaempferol (**h**), myricetin (**i**); flavanones-naringenin (**j**), hesperetin (**k**), taxifolin (**l**); hydroxybenzoic acids-gallic acid (**m**), *p*-hydroxybenzoic acid (**n**), quinic acid (**o**); hydroxycinnamic acids–caffeic acid (**p**), caftaric acid (**q**), *p*-coumaric acid (**r**); stilbenoids–*trans*-resveratrol (**s**), *trans*-piceatannol (**t**), *trans*-piceid (**u**).

The main *V. vinifera* groups of compounds are phenolic constituents and aromatic acids. Stilbenoid compounds have also been identified in relatively large amounts. It is important to note that there are qualitative differences in the bioactive compounds, which depend on the *V. vinifera* plant organ. The chemical structures of the main compounds and the composition diversity for individual parts of the plants are demonstrated in Figure 1 and Table 1.

The dominant groups of compounds in *V. vinifera* red fruits are anthocyanins (e.g., maldivin, cyanidin, petunidin and peonidin), in both red and white: procyanidins (e.g., B1, B2, B3, B4, C1 and T2), flavonoids (e.g., quercetin and kaempferol), phenolic acids (e.g., gallic acid and coumaric acid) and stilbenoids (e.g., *trans*-resveratrol and piceatannol) (Table 1) [5,16,29].

The major constituents of *V. vinifera* seeds are polyphenols (60–70%), which are mainly the flavan-3-ol derivatives, primarily catechin, epicatechin and epicatechin-3-*O*-gallate. Procyanidins are also found in *V. vinifera* seeds (e.g., procyanidin B1, B2, B3, B4, C1 and T2). The *V. vinifera* seeds are also a rich source of fatty acids, vitamins and minerals (Table 1) [5,16,32]. The *V. vinifera* seed oil is a raw material with a high nutritional value. It is characterized by the rich fatty acid profile, which is particularly plentiful in linoleic acid (≈70%). Other fatty acids presented in seed oil are oleic (≈15%), palmitic (≈7%), and stearic (≈3%) acids. The *V. vinifera* seed oil is also a rich source of tocopherols and tocotrienols. The most abundant vitamin E isomers are γ-tocotrienol followed by α-tocotrienol. The hydrophilic constituents, such as flavonoids, phenolic acids and tannins, are also found in *V. vinifera* seed oil. The composition of seed oil depends on the environmental factors of vine variety and the seed maturation degree [39,40,41]. Active ingredients present in seed oil can be extracted by various organic solvents. However, the residues of these solvents make the extracts obtained less valuable for the pharmaceutical or cosmetic industry because of their potentially hazardous properties [42]. What is more, the use of high temperatures in extraction processes may cause thermal degradation of active substances. Supercritical carbon dioxide (CO_2_) extraction can be an alternative to conventional extraction with organic solvents. The density of CO_2_ in the supercritical state is similar to organic solvents, which means that substances dissolve in them as in liquids, while the viscosity and surface tension are much lower. This allows for better penetration of the raw material. Since the obtained extracts do not contain chemical impurities, the process is environmentally safe and used CO_2_ can be recycled and reused. CO_2_ is considered a completely safe, non-toxic, non-flammable and relatively cheap eluent [43,44]. Grape seed oil constituents as non-polar compounds can be efficiently obtained using supercritical CO_2_ as the extractor. Wenli et al., in their work, applied this solvent-free, green process to extract resveratrol [45], while Passos et al. evaluated the process conditions to obtain selected fatty acids and tocopherol [46].

Flavonols are the most commonly occurring phenolic class in *V. vinifera* leaves, followed by stilbenoids, flavan-3-ols, anthocyanins, hydroxycinnamic and hydroxybenzoic acids [5] (Table 1).

The dominant phenolic classes presented in *V. vinifera* stems and canes are stilbenes (e.g., *trans*-resveratrol, (+)-*trans*-*ε*-viniferin and isohopeaphenol), followed by flavan-3-ols (e.g., catechin, procyanidin B, epicatechin and prodelphinidin A), hydroxybenzoic acids (e.g., gallic acid and protocatechuic acid) and hydroxycinnamic acids (e.g., sinapic acid and ferulic acid) [5,38] (Table 1).

The *V. vinifera* roots mainly contain stilbenoid compounds like hopeaphenol, ampelopsin A, vitisin A, isohopeaphenol and *trans*-resveratrol. There are no reports related to the other compounds present in the *V. vinifera* roots [5].

The sustainable exploitation of biological resources such as plant materials leads to the reduction of environmental impacts. The wastes obtained from plants could be potentially applied in the food industry to improve the nutritional food quality due to the presence of lipids, proteins and fiber or in the pharmaceutical field to retain the bioactive molecules [47,48]. Wastes obtained from *V. vinifera*, such as shoots, canes, stems, leaves and pomace are a rich source of bioactive compounds, e.g., stilbenes, flavan-3-ols, flavonols, hydroxybenzoic or hydroxycinnamic acids. Moreover, applications of *V. vinifera* waste-derived bioactive compounds have been found in cosmetic formulations [49].

## 3. Methods of Extraction and the Identification of Selected Groups of *V. vinifera* Metabolites

The standard process of the efficient extraction of *V. vinifera* active metabolites is based on low-temperature processes in hydroalcoholic solutions. Ultrasound-assisted extraction (UAE) is commonly used as the process does not require the application of high temperatures, which is extremely important for heat-sensitive compounds [50]. Tannins can be extracted with the highest efficiency by the SPE (solid phase extraction) method. However, standard extraction with methyl alcohol and ethyl acetate 1:1 (*v/v*) is also commonly applied [31]. Stilbenoids are commonly extracted in aqueous–alcoholic conditions or aqueous–acetone solutions [51]. Oligomeric tannins, which are quite demanding metabolites according to their large molecular mass, are extracted by multistep processes, including lyophilisation, extraction, evaporation, liquid-liquid crude fractionation, solubilisation in water and second extraction. These multistep processes are aimed at obtaining tannins at high levels of purity because of the presence of particular lipophilic metabolites known as ballast compounds. Thus, liquid-liquid crude fractionation allows the removal of lipids and pigments [52,53].

The identification of the grape metabolites is practised mostly by the UPLC-MS method. Chromatographic and mass spectra data are given for analysed compounds [31]. Table 2 presents an overview of the methods of *V. vinifera* for the extraction and quantification of selected metabolite groups.

## 4. The Position of *V. vinifera* in the Official Documents

### 4.1. EMA–HMPC

In 2010, the European Medicines Agency (EMA), by the de*cis*ion of the Committee on Herbal Medicinal Products (HMPC), approved the use of *Vitis viniferae folium* (*V. vinifera* leaves) for the treatment of chronic venous insufficiency associated with swollen legs, varicose veins, a feeling of heaviness, tiredness, tension and pain in the calves. *V. vinifera* leaf preparations may also be helpful in the heaviness of legs that is associated with minor blood circulation problems in the veins or in the itching and burning sensations related to haemorrhoids. This opinion is based on scientific studies proving the effectiveness and safety of these preparations [11].

### 4.2. EFSA

The European Food and Safety Authority (EFSA) has approved *V. vinifera* seed and dry extracts as a water-flavouring additive for animals (except dogs) in a specified concentration. However, the EFSA concluded that the application of *V. vinifera* for the improvement of circulation or the reduction of swelling in the legs is not sufficient, and further analysis is required [13].

### 4.3. FDA

The U.S. Food and Drug Administration (FDA) has approved the use of *V. vinifera* fruits and leaves and their extracts as a component of the human diet. However, there are studies proving that *V. vinifera* var. Reiber and var. Tokays can cause an allergic reaction. The FDA has also listed *V. vinifera* extract as an ingredient in wound dressings [12].

### 4.4. CosIng

According to the CosIng database, *V. vinifera* fruit (*Vitis viniferae fructus*) can be used as a skin-conditioning agent. The seeds of *V. vinifera* (*Vitis viniferae semen*) are used as skin-protecting and conditioning components. The seeds have also shown indications of being anti-seborrheic, antimicrobial and antioxidant ingredients. The *V. vinifera* seed oil can be used as an emollient. The roots (*Vitis viniferae radix*) have skin-conditioning properties. The *V. vinifera*e *cauli* (shoot) have skin-protecting and antioxidant properties. *V. vinifera* leaves (*Vitis viniferae folium*) are highly valued in cosmetic production as a skin-conditioning agent or fragrance [20]. A detailed description of *V. vinifera* with its functions based on the CosIng database is presented in Table 3.

## 5. *V. vinifera* as the Ingredient of the Cosmetic Formulation

According to the FDA’s Voluntary Cosmetic Registration Program (VCRP), from 2012, *V. vinifera* seed extract was used in 495 cosmetic formulations. *V. vinifera* fruit extract was used in 238 cosmetic formulations. *V. vinifera* leaf extract was reported to be used in 80 cosmetic formulations. The remaining *V. vinifera*-derived ingredients were used in fewer than 15 cosmetic formulations [16].

Nowadays, the production of cosmetics based on *V. vinifera* extracts is particularly popular in the countries of southern and central Europe, the United States, China and South Korea. It could be noted that *V. vinifera* extracts or oil demonstrate particular moisturising abilities as well as anti-ageing properties, which are confirmed by the increasing number of cosmetics containing these raw materials. Table 4 presents examples of cosmetic products containing *V. vinifera*-derived ingredients.

## 6. Biological Activities of *V. vinifera* Confirmed by Scientific Reports with a Direct Application in Cosmetology

### 6.1. Anti-Aging and UV-Protection Activities

Letsiou et al. [23] evaluated the effect of *V. vinifera* leaf extracts on UV-stressed human dermal fibroblasts. Fresh leaves of *V. vinifera* var. Athiri extracted with a solvent system of glycine-H_2_O (4:1) were used. Primary normal human fibroblasts (NHDF) were isolated from adult human skin, incubated with *V. vinifera* extract (0.1 μg/mL) and incubated for 48 h. Cells were washed twice with phosphate-buffered saline (PBS) and exposed to UVA light. During analysis, the significant induction of sirtuin 1 (SIRT1) and heat shock protein 47 (HSP47) is demonstrated with the presence of *V. vinifera* extract under normal and UV conditions. In addition, DNA methylation changes were observed, which appear to have been induced by the *V. vinifera* extract. The results of the investigation clearly prove the protective effect of the *V. vinifera* extract, which is possibly associated with a *trans*criptional regulation of skin anti-ageing genes [23] (Table 5).

Cefali et al. [57] investigated the effectiveness of *V. vinifera* var. Benitaka skin extract with regard to sun protection, antioxidative activity and skincare formulation stability. The skins were extracted in ethanol and standardised with HPLC-DAD for the determination of flavonoid content. The results of the cell viability test showed that the extract had no effect on cell viability. In order to determine the effectiveness of the extract as a sun filter, in vitro SPF was determined and was equal to 18.56. The UVA protection factor determined by the spectral *trans*mittance was 3.17, with a critical wavelength of 318 nm and a UVA/UVB rate of 0.9. The antioxidant activity was tested by DPPH and ABTS assays. In both assays, the extract exhibited antioxidant activity, reducing the DPPH and ABTS concentrations by 92.08% and 86.85%, respectively. The properties of the extract observed within a stable oil-in-water (O/W) emulsion support the potential use of the formula as a sunscreen. The emulsion was odourless, glossy and light pink with a characteristic desirable for skincare formulations (pH: 5.50, density: 1.001 g/mL, viscosity: 13,000.35 cP) [57] (Table 5).

### 6.2. Anti-Inflammatory Activity

Sangiovanni et al. [58] evaluated the ability of an aqueous extract of the leaves of *V. vinifera* var. Teinturiers inhibit inflammation in human keratinocytes (HaCaT cells) caused by the mediators of inflammation or oxidative stress, which are released in psoriasis. Human keratinocytes were cultured using Dulbecco’s Modified Eagle Medium (DMEM) supplemented with penicillin, streptomycin, L-glutamine, and 10% heat-inactivated Fetal bovine serum (FBS) and cultured in twenty-four-well plates. It was then treated with inflammatory mediators: tumour necrosis factor-*α* (TNF -*α*) and lipopolysaccharide (LPS). Human keratinocytes cells were plated and *trans*fected with plasmid NF-kB-LUC (nuclear factor Kappa B luciferase) or native IL-8-LUC (IL-8 Luciferase) promoter, which contains sequences responsive to several *trans*cription factors, both at 250 ng per well. The cells were treated with increasing concentrations of *V. vinifera*-leaf extract in the presence of inflammatory mediators and after the luciferase assay was performed. It was demonstrated that the extract inhibited the interleukin-8 (IL-8) secretion induced by TNF-*α* (IC_50_ = 2.60) or LPS (IC_50_ = 14.04). In addition, it was also associated with the inhibition of the nuclear factor- κB (NF-κB)-driven *trans*cription which indicates the presence of the anti-inflammatory properties of grape extracts [58] (Table 5).

**Table 5:** Biological activity of *V. vinifera* with the direct application in cosmetology.

Biological Activity	Tested Plant Material	Mechanism of Action	References
Anti-ageing activity	*V. vinifera* leaf extract	-the stimulation of SIRT 1 and HSP 4 genes	[23]
UV-protection activity	*V. vinifera skin* extract	-possessing skin-protecting activity against sun rays	[57]
Antioxidant activity	*V. vinifera* fruit extract *V. vinifera* skin extract *V. vinifera* leaf extract	-free oxygen radical scavenging	[21,59] [19] [59]
	*V.vinifera* stem extract		[60]
	*V. vinifera* pomace extract	-oxidation of human LDL lipoproteins -influence on lipid peroxidation	[61]
Anti-inflammatory activity	*V.vinifera* leaf extract	-inhibition of pro-inflammatory cytokines	[58]
Skin-whitening activity	*V.vinifera* leaf extract	-tyrosinase inhibition	[22]
	*V. vinifera* cane extract	-tyrosinase inhibition	[50]

### 6.3. Skin-Whitening Activity

Lin et al. [22] tested the effectiveness of *V. vinifera* leaf extract on the tyrosinase inhibitory activity. The presence of gallic acid, chlorogenic acid, epicatechin, rutin and *trans*-resveratrol in the extracts was detected with the HPLC method. It was demonstrated that *V. vinifera* leaf extract reduced the tyrosinase activity in a dose-dependent manner (IC_50_ = 3.84 mg/mL). The kinetic study showed the tyrosinase inhibitory activity using a competitive mechanism [22] (Table 5).

Malinowska et al. [50] investigated the rejuvenating effect of five selected varieties of *V. vinifera* (Villard Noir, Sauvignon, Savagnin, Riesling and Magdeleine Noire des Charentes) cane extracts by tyrosinase inhibition and the delaying of cell ageing. The skin whitening potential of *V. vinifera* cane extracts was compared to pure *trans*-resveratrol and *ε*-viniferin. The HPLC-MS analysis determined the main polyphenols presented in the ethanol-water (60/40 *v/v*) extract, namely catechin, epicatechin, piceatannol, *trans*-resveratrol, ampelopsin, *ε*-viniferin, hopeaphenol, isohopeaphenol, miyabenol C and vitisin B. The SIRT1 activity was determined using the SIRT1 assay kit. Most of the extracts showed relatively high SIRT activation. Among all the varieties, Riesling was the most potent, with 171% SIRT activation. The tyrosinase inhibition was performed with a tyrosinase inhibition assay. All the tested extracts are relatively efficient tyrosinase inhibitors. The highest results were obtained for *ε*-viniferin (76%) and *trans*-resveratrol (75%). Riesling and Villard Noir extracts showed the highest inhibition activity (62.5% and 58.5%) [50] (Table 5).

## 7. Biological Activities Confirmed by Scientific Reports with a Potential Application in Cosmetology

### 7.1. Antioxidant Activity

Antioxidant activity plays a significant role in the maintenance of good skin condition as well as in the prevention of numerous skin diseases and dysfunction. The main protective mechanisms of antioxidative molecules contained in grape extracts are free radical scavenging abilities. This simple mechanism ensures DNA damage repair, the modulation of gene expression in proliferation, metabolism, and cell survival, as well as the antioxidant defence [62]. It was proven that grape phytochemicals’ in vivo molecular mechanisms lead to health promotion by avoiding oxidative stress-related pathologies.

Zeghad et al. [21] evaluated the antioxidant activity of *V. vinifera*, Punica granatum, Citrus aurantium and Opuntia ficus indica fruits. The fruits were tested by using three different SET-based assays (ABTS, FRAP, DPPH) and a hydrogen-atom *trans*fer-based assay (ORAC). The results indicated that among all four tested fruit extracts, the highest antioxidant capacity was showed for *V. vinifera* based on the applied methods (IC_50_ (50% inhibitory concentration) = 0.040 mg/mL, 0.98 mg/mL, 0. 270 mg/mL and 2036 μM TE/g, respectively) [21] (Table 5).

Tzanova et al. [19] evaluated the antioxidant activity of the commercial *V. vinifera* skin extracts of different red varieties obtained from separate Bulgarian regions. The antioxidant potential and the total phenol content were measured by UV methods. All the tested extracts have a similar radical scavenging capacity ranging from 23.2 ± 1.7 to 48.7 ± 5.1 mmol/kg Trolox equivalent (TE), depending on the variety. The highest antioxidant activity was observed for the Syrah variety (48.7 ± 5.1 mmol/kg) from the Mogilovo vineyard. The total phenolic content ranged from 33.4 ± 4 in Merlot to 202 ± 19 mmol/kg gallic acid equivalent (GAE) in the Syrah variety [19].

Zielonka-Brzezicka et al. [59] tested the antioxidant activity of fresh and frozen *V. vinifera* fruits and leaves of an unspecified red variety. The antioxidant activity was established by ABTS and DPPH assays in ethanol, methanol, isopropanol and water extracts. The methanolic extracts of fresh leaves showed the highest activity in the DPPH assay: 3.12 AAE (ascorbic acid equivalent, mg AA/g of raw material). Moreover, the highest antioxidant capacity was indicated for the frozen leaves extracted with isopropanol in the ABTS assay (26.94 AAE) [59] (Table 5).

Llobera [60] confirmed the antioxidant activity of *V. vinifera* stems. Some 80% and 70% acetone extracts of the red variety Manto Negro and white variety Prensal Blanc were used. The free radical scavenging activity was determined by the DPPH assay. The values of EC_50_ (half maximal effective concentration) of extracts obtained from red-grape variety Manto Negro extracts were 0.14 g dm (dry matter)/g DPPH and 0.20 g dm/g DPPH for the acetone and the ethanol extracts, respectively. The white grape variety Prensal Blanc extracts showed 0.26 g dm/g DPPH and 0.37 g dm/g DPPH, respectively. Studies showed that the antioxidant activity of *V. vinifera* stem extracts significantly correlated with the total content of polyphenols and flavanols [60] (Table 5).

Chidambara et al. [61] evaluated the antioxidant activity of *V. vinifera* pomace ethyl acetate, methanol and water extracts using different methods. The methanol extracts showed the highest antioxidant activity (87%) in the DPPH assay. Ethyl acetate and water extracts showed 76% and 21.7%, respectively. The methanol extract demonstrated the strongest activity and was selected for further analysis using the thiobarbituric acid method, hydroxyl scavenging activity and LDL oxidation. The methanolic extracts showed inhibition levels of 71.7, 73.6, and 91.2%, respectively. The in-vivo study demonstrated that treatment with a single dose of 1.25 mg/kg of CCl_4_ decreases the activity of peroxidase (89%), catalase (81%) and superoxide dismutase (49%) in albino rats. The pre-treatment of the rats with 50 mg/kg grape pomace methanolic extract followed by the treatment with CCl_4_ resulted in catalase, peroxidase and SOD restoration at the level of 43.6, 54.0 and 73.2%, respectively. The histopathological studies of the liver of the different groups confirm the protective effect of the *V.vinifera* pomace methanolic extract, which contributed to the restoration of normal liver structure [61] (Table 5).

### 7.2. Antimicrobial Activity

Oliveira et al. [63] tested the antimicrobial activity of *V. vinifera* pomace extracts of Merlot and Syrah varieties. The extracts were obtained by a supercritical CO_2_ extraction method, and CO_2_ was added with co-solvent (ethanol) extraction at pressures of up to 300 bar and temperatures of 50 and 60 °C. The constituents of the extracts were identified using the HPLC method. The dominant compounds were gallic acid, *p*-hydroxybenzoic acid, vanillic acid and epicatechin. The antibacterial activity and antifungal activity were assessed against the Bacillus cereus, Escherichia coli, Staphylococcus aureus, Pseudomonas aeruginosa bacterial strains and the Candida albicans, Candida parapsilosis and Candida krusei fungal strains. The supercritical fluid extracts showed high antimicrobial activity (inhibition > 9 mm), particularly against Gram-positive bacterial strains. The SFE SC-CO_2_ (supercritical fluid extraction obtained by supercritical CO_2_) extracts of the Merlot variety were effective against C. albicans and C. krusei with MIC (minimum inhibitory concentration) of 500 µg/mL [63] (Table 6).

Filocamo et al. [64] evaluated the antimicrobial activity of white grape juice extract derived from a mixture of white grape juice containing Catarratto, Grillo and Insolia *V. vinifera* varieties. The antimicrobial activity was tested against *S. aureus*, *Listeria monocytogenes*, *Staphylococcus epidermidis*, *Enterococcus hirae*, *Streptococcus pneumoniae*, *Bacillus subtilis*, *Streptococcus pyogenes*, *Enterococcus durans*, *Streptococcus mutans*, *Moraxella catarrhalis* Gram-positive bacteria strains, *Salmonella typhi*, *Serratia marcescens*, *E. coli*, *P. aeruginosa*, *Proteus mirabilis*, *Klebsiella pneumoniae* Gram-negative bacteria strains and *Aspergillus niger* and *C. albicans* fungal strains. The extract was obtained by passing the juice through the must-mute columns equipped with adsorbent resins, which retain polyphenols. The molecules were eluted with 4% NaOH and passed through the cationic resins. The products were then collected, filtered and sprayed in order to obtain a dry powder. The dominant polyphenols determined in *V. vinifera* juice extract were quercitin-3-glucuronide, procyanidin B1, quercetin-3-glucoside, catechin and *trans*-coutaric acid. The *V. vinifera* juice extract inhibited all tested Gram-positive bacteria (MIC = 3.9–1000 μg/mL^−1^. The best results were observed for *S. aureus* [64] (Table 6).

Yadav et al. [65] accessed the antibacterial and antifungal activities of the Sharad variety of *V. vinifera* seedless-fruit-skin extracts against antibiotic-resistant pathogenic bacteria and toxin-producing moulds. Among the tested bacteria strains were *Enterococcus faecallis, S. aureus, Salmonella typhimurium, Enterobacter aerogenes* and *E. coli*. The antifungal activity was tested for the following strains: *Penicillum expansum, Penicillum chrysogenum, A. niger* and *Aspergillus versicolor*. The *V. vinifera* skin was extracted using different solvents: water, acetone, ethanol and methanol. The antibacterial activity was determined by the agar well diffusion method. The antifungal activity was evaluated as a percentage of conidia germination inhibition. The methanolic extracts possessed strong antibacterial and antifungal activity. The maximum zone of inhibition was determined for *S. aureus* (22 mm), followed by *E. faecalis* (18 mm) and *E. aerogenes* (21 mm) [65] (Table 6).

### 7.3. Anti-Inflammatory Activity

Di Lorenzo et al. [7] tested the anti-inflammatory activity of extracts from raisins of different varieties: Early Gold (Portugal) and Sultana (Turkey). Attention was focused on the interleukin (IL-8) and nuclear factor (NF)-κB pathways. The composition of raisin extracts was evaluated by the HPLC-DAD method and screened for the ability to inhibit IL-8 release induced by a tumour necrosis factor (TNF-*α*) and promoter activity in human gastric epithelial cells. The Turkish variety (Sultana) inhibited the release of IL-8 affected by the impairment of promoter activity. The researchers also tested the seed extract, which showed slightly higher inhibitory activity against IL-8 and (NF)-κB than the raisin extract. The results suggest that the consumption of selected raisins (for instance, the Sultana variety) could be beneficial against gastric inflammatory diseases [7] (Table 6).

Chopra and Geetha [66] studied the anti-inflammatory effect of *V. vinifera* seed extract using the albumin denaturation assay. The extract was tested in different concentrations from 10–50 μg/mL. The results showed that the *V. vinifera* seed extract possessed better anti-inflammatory activity in comparison to diclofenac sodium used as a reference. The extract also had fewer side effects. It is suggested that polyphenols are responsible for this effect [66] (Table 6).

## 8. The Applications of *V. vinifera* In Vitro Cultures in Cosmetology

In recent years, natural cosmetics with innovative ingredients have been in demand. The particular interest of the cosmetic industry is focused on *V. vinifera* stem cells, which have applications mainly in anti-ageing creams and essences [1]. The Mibelle Biochemistry company (Switzerland) has developed a new biotechnology technique under the name PhytoCellTech™, which is used to generate plant stem cells. The growth of *V. vinifera* callus cells was induced under special conditions. The undifferentiated callus cells, i.e., stem cells, are involved in further cultivation in special bioreactors to obtain a sufficient amount of plant cells. The technology is highly sustainable and enables the production of large amounts of high-quality active ingredients. The PhytoCellTech™ Solar Vitis is a stem cell obtained from the Gamay Teinturier Fréaux variety of *V. vinifera* of French origin, which was characterised as a high-polyphenol line [67].

**Figure 2:**
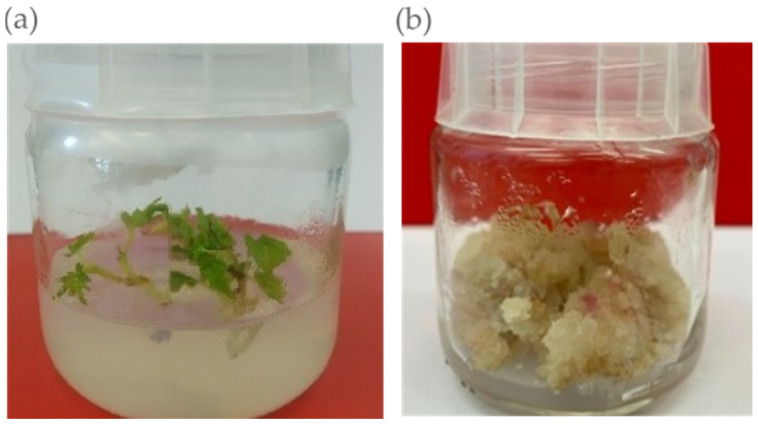
*V. vinifera* in vitro cultures: (**a**) shoots of Jutrzenka variety; (**b**) callus of Cabernet Cortis variety [unpublished].

Due to the recent high interest in in vitro cultures, including at the Faculty of Pharmacy of Jagiellonian University *Collegium Medicum*, Cracow (Poland), the *V. vinifera* in vitro culture of different varieties has been performed (Figure 2). The aim of the study is the qualitative and quantitative analysis of the extracts obtained from *V. vinifera* in vitro cultures followed by the determination of the total phenolic content (Folin-Ciocalteu method), the evaluation of the antioxidant activity using different methods (e.g., DPPH, ABTS, FRAP) and the future application of the most potent varieties in cosmetic formulations [unpublished].

Before for production of secondary metabolites, Bonello et al. [68] studied the *V. vinifera* var. Ġellewża callus cultures. Callus was incubated in an MS medium with plant growth regulators (PGRs) (6-benzylaminopurine (BAP); 3-indoleacetic acid (IAA); kinetin (KIN); 1-naphthaleneacetic acid (NAA); IAA+BAP; IAA+KIN; NAA+BAP; NAA+KIN) to determine its best combination needed for the production of metabolites. Some 0.5 g aliquots of callus extracts were analysed by UV-Vis spectrophotometry and HPLC method. The best callus production was obtained when the MS medium was enriched with IAA and IAA+BAP. The high content of flavonoids, mainly anthocyanins, was associated with the presence of cytokinins (especially BAP). Catechin, luteolin, myricetin, naringenin and quercetin-3-*O*-glucoside were identified among the flavonoids. *Trans*-resveratrol and polydatin were the most abundant among the stilbenoid compounds. Two coumarin derivatives were identified (aesculetin and aesculin) [68].

Mewis et al. [69] studied the production of polyphenolic compounds in callus cultures of *V. vinifera* var. Gamay Fréaux. The cell cultures were cultivated on B5 medium and were transferred to the fresh sterile medium after twenty-eight days. The red callus cultures were selected for future cultivation. The cultures were transferred every three weeks to fresh Erlenmeyer flasks containing the B5 medium. The obtained samples were frozen and lyophilised. The HPLC method was used for the analysis of 70% methanol extracts. The HPLC analysis revealed the presence of phenolic acid derivatives such as 3-*O*-glucosylresveratrol and 4-(3,5-dihydroxyphenyl)-phenol and cinnamoyl derivatives, including cyanidin 3-*O-p*-coumaryl glucoside and peonidin 3-*O-p*-coumaryl glucoside. The major anthocyanins identified in callus cultures were cyanidin 3-*O*-glucoside and peonidin 3-*O*-glucoside. The anthocyanins levels were significantly increased after cultivation for four days in the new medium [69].

## 9. Safety of Use

According to the Voluntary Cosmetic Registration Program (VCRP), data obtained from FDA in 2012, ingredients derived from *V. vinifera* can be used in different cosmetics formulations, depending on the raw material, but in relatively low concentrations. For instance, *V. vinifera* leaf extract could be present in leave-on formulations at levels of up to 3%. *V. vinifera* fruit extract and *V. vinifera* juice could be used in skin-cleansing products and masks at levels of up to 2%. Other *V. vinifera*-derived ingredients are included at levels of up to 1% in formulations. Grape skin extract contains enocianine, which is approved as a food colour additive with no required certification. According to the evaluation of the Joint Food and Agriculture Organization of the United States and the World Health Organization (FAO/WHO) Expert Committee on Food Additives (JECFA), the acceptable daily intake (ADI) of grape skin extract varies from 0–2.5 mg/kg bw (body weight) [2,16].

The cosmetics formulations containing the ingredients from *V. vinifera* could be applied to the eye area or mucous membranes and could also accidentally be ingested. Furthermore, the majority of *V. vinifera* extracts from different parts of the plant (fruit, leaf and seed), as well as *V. vinifera* juice and *V. vinifera* fruit water extracts presented in the cosmetics, could possibly be inhaled [16].

### 9.1. Skin Irritation and Sensitisation

In a dermal irritation test on human skin, products containing 3% *V. vinifera* fruit extract are non-irritant. According to the Epiderm MTT viability assay, products containing 10% of *V. vinifera* fruit extract are either non-irritant or minimally irritant. In in vitro assay, the hydrolysed grape skin did not demonstrate the stimulating potential of the monocytes and macrophages mediated cellular immune response. As reported by the human two-week use study, the product containing 0.15% of *V. vinifera* seed extracts was also non-irritant. In a clinical test conducted using patch tests, extracts from *V. vinifera* fruits, juice and seeds at a maximum concentration of 1% also did not demonstrate any irritating or sensitising potential [16].

### 9.2. Eye Irritation

The ocular irritation of a product containing 3% *V. vinifera* fruit extracts is predicted to be minimal in an EpiOcular assay. The ocular irritation potential was evaluated using the ocular irritation test for a single sample of a product with 3% *V. vinifera* fruit water extract. The irritation Draize equivalent (IDE) score ranged from 4.5–6.4. A product containing 10% *V. vinifera* fruit extract was classified as a non/minimal irritant. Hydrolysed grape skin extracts are predicted to be ocular non-irritating in a cytotoxicity assay. In in vitro testing, a product containing 0.15% *V. vinifera* seed extracts was found to be a mild ocular irritant [16].

## 10. Conclusions

*V. vinifera* is one of the most popular fruit crops around the world. It is a useful species which is cultivated within all continents, especially in Europe, the Middle East and Asia. Its valuable properties have been known since ancient times and used for a number of ailments, including cancer, eye infection, sore throat and nausea [4,28,70]. Currently, *V. vinifera* is intensively exploited primarily in terms of sustainable development. The waste matter of the vine grapes (e.g., stems, pomaces and seeds) are desirable raw materials which contain valuable bioactive compounds [49]. Recently, *V. vinifera* has been an extremely preeminent plant used in the food and pharmaceutical industries.

Numerous scientific studies have proven the valuable chemical composition of *V. vinifera*, which is dominated by phenolic compounds. The main group of metabolites present in *V. vinifera* are flavonoids, stilbenoids, phenolic acids, anthocyanins, catechin derivatives, procyanidins, fatty acids and vitamins [5,30]. The obtaining and the quantitative analysis of grape extracts are well established in the literature, and the most challenging metabolites for quantification and purification are condensed tannins [54].

Despite the properties of *V. vinifera*, it is not mentioned in any pharmacopeia. Nevertheless, there are monographs with a positive opinion provided by respected organisations such as the EMA, the FDA and the EFSA [11,12,13].

In the food industry, *V. vinifera* is used mainly to produce wine, juice and raisins [18].

The phytochemical composition of *V. vinifera* determines the antioxidant, antibacterial and anti-inflammatory activities as well as the cardioprotective, neuroprotective and hepatoprotective properties. These *V. vinifera* activities are especially important for the pharmaceutical industry [6,7,8,9,10].

Due to the widespread application of *V. vinifera* in the cosmetics industry, it deserves special attention. Raw materials obtained from *V. vinifera* are highly valued in cosmetics, particularly due to their antioxidant, anti-ageing, skin-whitening and UV-protection properties. The proven safety of *V. vinifera* also contributes to its extensive use. There is a wide range of cosmetics based on *V. vinifera*-derived ingredients [20]. Nowadays, the production of cosmetics based on *V. vinifera* is particularly popular in the countries of southern and central Europe, the United States, China and South Korea.

*V. vinifera* is also a research subject in terms of biotechnological studies. There is growing interest in *V. vinifera* in vitro stem cells as well as tissue cultures [1,67]. It is expected that *V. vinifera* in vitro culture extracts will be proposed as innovative and effective cosmetic ingredients in the future.

## Figures and Tables

**Table 1:** *V. vinifera* chemical composition diversity for individual plant parts.

Plant Part	Compounds	References
Fruits	**Anthocyanins:** cyanidin 3-*O*-(6″-*p*-coumaroyl-glucoside), cyanidin 3-*O*-glucoside, delphinidin 3-*O*-(6″-acetyl-glucoside), delphinidin 3-*O*-glucoside, malvidin 3-*O*-(6″-acetyl-glucoside), malvidin 3-*O*-(6″-*p*-coumaroyl-glucoside), malvidin 3-*O*-glucoside, peonidin 3-*O*-(6″-*p*-coumaroyl-glucoside), peonidin 3-*O*-glucoside, petunidin 3-*O*-(6″-*p*-coumaroyl-glucoside), petunidin 3-*O*-glucoside **Ellagitannins:** castalagin, vescalagin, grandinin, roburin A, roburin B, roburin C, roburin D, roburin E, and acutissimins A and B **Flavan-3-ols:** procyanidin B1, procyanidin B2, procyanidin B3, procyanidin B4, procyanidin B1 3-*O*-gallate, procyanidin B2 3-*O*-gallate, procyanidin C1, procyanidin T2 **Flavonols:** quercetin 3-*O*-galactoside, quercetin 3-*O*-glucuronide, quercetin 3-*O*-rutinoside, isorhamnetin 3-*O*-glucoside, kaempferol 3-*O*-galactoside, kaempferol 3-*O*-glucoside **Hydroxycinnamic acids:** caffeoyl tartaric acid, *cis*-caffeoyl tartaric acid, *trans*-caffeoyl tartaric acid, *p*-coumaroyl tartaric acid, *trans*-*p*-coumaroyl tartaric acid **Stilbenoids:** monomeric (resveratrol, *trans*-resveratrol, resveratrol-3-*O*-glucoside, *trans*-resveratrol-3-*O*-glucoside, piceatannol) **Vitamins and minerals:** vitamin C, vitamin B1, vitamin B2, vitamin B3, vitamin B5, vitamin B6, vitamin B7, vitamin B9, myo-inositol; potassium (K), sulfur (S), copper (Cu), phosphorus (P), magnesium (Mg), iron (Fe), calcium (Ca), manganese (Mn), zinc (Zn), boron (B)	[5,16,29,30,31]
Skins	**Anthocyanins:** delphinidin-3,5-diglucoside, delphinidin-3-monoglucoside, malvidin-3,5-diglucoside, cyanidin-3-monoglucoside, petunidin-3-monoglucoside, pelargonidin-3-monoglucoside, peonidin-3-monoglucoside, malvidin-3-monoglucoside, delphinidin-3-(6-acetyl)-glucoside, cyanidin-3-(6-acetyl)-glucoside, petunidin-3-(6-acetyl)-glucoside, delphinidin-3-(6-caffeoyl)-glucoside, cyanidin-3-(6-caffeoyl)-glucoside, peonidin-3-(6-caffeoyl)-glucoside, malvidin-3-(6-acetyl)-glucoside, petunidin-3-(6-caffeoyl)-glucoside, delphinidin-3-(6-coumaroyl)-glucoside, peonidin-3-(6-caffeoyl)-glucoside, malvidin-3-(6-caffeoyl)-glucoside, cyanidin-3-(6-coumaroyl)-glucoside, petunidin-3-(6-coumaroyl)-glucoside, peonidin-3-(6-coumaroyl)-glucoside, malvidin-3-(6-coumaroyl)-glucoside **Fatty acids:** linoleic acid, palmitic acid, myristic acid, *cis*-7-hexadecenoic fatty acid, stearic acid, oleic acid, *α*-linolenic acid, arachidic acid, behenic acid, lignoceric acid, saturated fatty acids, monounsaturated fatty acids, polyunsaturated fatty acid **Flavan-3-ols:** procyanidin B1, procyanidin B2, procyanidin B3, procyanidin B4, procyanidin B1 3-*O*-gallate, procyanidin B2 3-*O*-gallate, procyanidin C1, procyanidin T2 **Minerals:** potassium (K), sulfur (S), copper (Cu), phosphorus (P), magnesium (Mg), iron (Fe), calcium (Ca), manganese (Mn), zinc (Zn), boron (B)	
Seeds	**Carboxylic acids:** primaric acid, *p*-hydroxyphenylacetic acid **Fatty acids:** myristic acid, palmitic acid, *cis*-7-hexadecenoic fatty acid, margaric acid, stearic acid, oleic acid, linoleic acid, *α*-linolenic acid, arachidic acid, behenic acid, saturated fatty acids, monounsaturated fatty acids, polyunsaturated fatty acid **Flavan-3-ols:** procyanidin B1, procyanidin B2, procyanidin B3, procyanidin B4, procyanidin B1 3-*O*-gallate, procyanidin B2 3-*O*-gallate, procyanidin C1, procyanidin C2, epicatechin, catechin **Flavonols:** quercetin, quercetin-3-β-D-glucoside, quercitrin, myricetin **Hydroxybenzoic acids:** gallic acid **Hydroxycinnamic acids:** caffeic acid, coumaric acid, ferulic acid, fertaric acid **Stilbenoids:** monomeric–*trans*-resveratrol, dimeric–*trans-ε*-viniferin **Vitamins and minerals:** vitamin A, vitamin E; potassium (K), sulfur (S), copper (Cu), phosphorus (P), magnesium (Mg), iron (Fe), calcium (Ca), manganese (Mn), zinc (Zn), boron (B)	[5,16,32,33,34]
Leaves	**Anthocyanins:** delphinidin-3-*O*-glucoside, cyanidin-3-*O*-glucoside, petunidin-3-*O*-glucoside, peonidin-3-*O*-glucoside, malvidin-3-*O*-glucoside, petunidin-3-(6-*O*-acetyl)glucoside, peonidin-3-(6-*O*-acetyl)glucoside, malvidin-3-(6-*O*-acetyl)glucoside, cyanidin-3-(6-*O*-coumaroyl)glucoside, petunidin-3-(6-*O*-coumaroyl)glucoside, peonidin-3-(6-*O*-coumaroyl)glucoside, malvidin-3-(6-*O*-coumaroyl)glucoside **Coumarins:** aesculin, fraxin, aesculutin, umbelliferone **Flavan-3-ols:** gallocatechin, catechin, procyanidin A1, procyanidin B1, procyanidin B2, procyanidin B3, procyanidin B4, epicatechin, epigallocatechin, epigallocatechin gallate, gallocatechin gallate, epicatechin gallate, catechin gallate **Flavonols:** quercetin, quercetin-3-*O*-glucoside, kaempferol, myricetin, myricetin-3-*O*-galactoside, myricetin-3-*O*-glucuronide, myricetin-3-*O*-glucoside, quercetin-3-*O*-rutinoside, quercetin-3-*O*-galactoside, quercetin-3-*O*-glucoside, quercetin-3-*O*-glucuronide, myricetin-3-*O*-rhamnoside, quercetin-3-*O*-rhamnoside, kaempferol-3-*O*-galactoside, kaempferol-3-*O*-rutinoside, kaempferol-3-*O*-glucuronide, quercetin-3-(6-*O*-acetyl)glucoside, quercetin-3-(3-*O*-arabinosyl)glucoside, quercetin-3-(7-*O*-glucosyl)glucuronide, kaempferol-3-*O*-glucoside, kaempferol-3-*O*-xyloside, kaempferol-3-*O*-rhamnoside, isorhamnetin-3-*O*-galactoside, isorhamnetin-3-*O*-glucoside, quercetin-3-(6-*O*-rhamnosyl)galactoside, isorhamnetin-3-*O*-arabinose, isorhamnetin-3-*O*-glucuronide, isorhamnetin-3-*O*-rutinoside, isorhamnetin-3-(4-*O*-rhamnosyl)rutinoside, kaempferol-3-(6-*O*-coumaroyl)glucoside, kaempferol-3(7-*O*-glucosyl)galactoside, diquercetin-3-(3-*O*-glucosyl)glucuronide **Flavones:** apigenin-7-*O*-glucoside, luteolin-7-*O*-glucoside **Flavanones:** taxifolin, naringenin, hesperetin, eriodictyol-7-*O*-glucoside, naringenin-7-*O*-glucoside **Hydroxybenzoic acids:** quinic acid, gallic acid, vanilic acid, syringic acid, protocatechuic acid, *p*-hydroxybenzoic acid, gentisic acid, *γ*-resorcylic acid, ellagic acid **Hydroxycinnamic acids:** caftaric acid, caffeic acid, fertaric acid, 1-*O*-sinapoyl-β-D-glucose, 1-*O*-(4-Coumaroyl)-glucose, 1-caffeoyl-β-D-glucose, ferulic acid pentose, coutaric acid, chlorogenic acid, *p*-coumaric acid, ferulic acid, sinapic acid, cinnamic acid **Dihydrochalcones:** phlorizin **Stilbenoids:** monomeric (*trans*-astringin, *trans*-resveratroloside, *cis*-resveratrol-*O*-glucoside, *trans*-piceid, *cis*-astringin, *trans*-piceatannol, *cis*-resveratroloside, *cis*-piceid, *trans*-isorhapontin, *trans*-resveratrol, 2,4,6-trihydroxyphenanthrene-2-*O*-glucoside, *trans*-isorhapontigenin, *trans*-pinostilbene-4-*O*-glucoside, *cis*-resveratrol, *trans*-pterostilbene, *cis*-pterostilbene, *cis*-isorhapontigenin, *trans*-rhaponticin, *trans*-pinostilbene, *cis*-pinostilbene, dimeric (restrytisol A, pallidol, ampelopsin D, quadrangularin A, (+)-*cis*-*ε*-viniferin, (+)-*trans*-*ε*-viniferin, *trans*-*ω*-viniferin, *cis*-*ω*-viniferin, *trans*-*δ*-viniferin, *cis-δ*-viniferin, *trans*-*ε*-viniferin derivative (dimethylated), *trans*-*δ*-viniferin derivative (dimethylated)), trimeric (ampelopsin B, *trans*-miyabenol C, *cis*-miyabenol C, davidiol A, *α*-viniferin), tetrameric (isohopeaphenol, ampelopsin H, vaticanol C-like isomer, hopeaphenol)	[5,35,36,37]
Stems/canes	**Anthocyanins:** malvidin-3-*O*-glucoside, malvidin-3-(6-*O*-caffeoyl) glucoside, malvidin-3-*O*-rutinoside **Flavan-3-ols:** gallocatechin, epicatechin, catechin, procyanidin B1, procyanidin B2, procyanidin B3, procyanidin B4, procyanidin B1 3-*O*-gallate, procyanidin B2 3-*O*-gallate, procyanidin A1, procyanidin C1, procyanidin T2, epigallocatechin, prodelphinidin A-type, procyanidin dimer gallate, epicatechin gallate, catechin gallate **Flavonols:** quercetin, quercetin-3-*O*-glucoside, kaempferol, quercetin-3-*O*-rutinoside, quercetin-3-*O*-galactoside, quercetin-3-*O*-glucuronide, quercetin-3-*O*-rhamnoside, kaempferol-3-*O*-rutinoside, quercetin-3-*O*-arabinose, kaempferol-3-*O*-glucoside, dihydrokaempferol-3-*O*-rhamnoside, isorhamnetin-3-(6-*O*-feruloyl) glucoside **Flavanones:** taxifolin-*O*-pentoside, taxifolin-3-*O*-glucoside, taxifolin-3-*O*-rhamnoside **Hydroxybenzoic acids:** gallic acid, syringic acid, protocatechuic acid, *p*-hydroxybenzoic acid, vanillic acid, ellagic acid **Hydroxycinnamic acids:** caftaric acid, caffeic acid, ferulic acid, 1-*O*-(4-coumaroyl)-glucose, 1-caffeoyl-β-D-glucose, ferulic acid pentose, chicoric acid, *p*-coumaric acid, coutaric acid, sinapic acid **Stilbenoids:** monomeric (*trans*-astringin, *trans*-resveratrol, *trans*-resveratroloside, *trans*-resveratrol-2-*C*-glucoside, *trans*-resveratrol-10-*C*-glucoside, *trans*-resveratrol-*O*-glucoside, *cis*-resveratrol-*O*-glucoside, *trans*-piceid, *cis*-piceid, *trans*-piceatannol, *trans*-isorhapontigenin, *trans*-pterostilbene, *cis*-pterostilbene) dimeric (leachianol G, leachianol F, restrytisol A, pallidol, caraphenol B, quadrangularin A, (+)-*trans*-*ε*-viniferin, viniferifuran, diptoindonesin A, *trans*-*δ*-viniferin, *trans*-*ω*-viniferin, *trans*-scirpusin A, maackin A, malibatol A, viniferal, vitisinol, C, vitisinol E, ampelopsin A, ampelopsin D, ampelopsin F), trimeric (*trans*-miyabenol C, *cis*-miyabenol C, davidiol A, *α*-viniferin, ampelopsin B, ampelopsin C, ampelopsin E, viniferol D), tetrameric (hopeaphenol, isohopeaphenol, ampelopsin H, vitisifuran A-B, vitisin A (r2-viniferin), vitisin B (*r*-viniferin), vitisin C, viniferol A, viniferol B, viniferol C), hexameric (viniphenol A)	[5,38]
Roots	**Stilbenoids:** monomeric (*trans*-resveratrol, *trans*-picaetannol, *trans*-piceid, *cis*-piceid), dimeric (vitisinol B, viniferether A-B, ampelopsin A, pallidol, (+)-*trans*-*ε*-viniferin, *trans*-*ω*-viniferin, *trans*-*δ*-viniferin), trimeric (*trans*-miyabenol C, ampelopsin C, ampelopsin E, viniferol D) tetrameric (vitisin A-B, hopeaphenol, isohopeaphenol, viniferol E, wilsonol C, heyneanol A, stenophellol C)	[5]

**Table 2:** Methods of the extraction and identification of *V. vinifera* bioactive metabolites.

Compound Group/ Extracted Raw Material	Extraction Conditions/ Purification	Analysis Method	References
Ellagitannins (castalagin, vescalagin, grandinin, roburin A, roburin B, roburin C, roburin D, roburin E and acutissimins A and B)/oak-aged wine	Solid phase extraction (SPE)/a combined elution with methanol and ethyl acetate (1:1 *v/v*)	HPLC-DAD-ESI-MS/MS	[31]
Oligomeric tannins (epicatechin vanillate)/grape seed and wine	Lyophilisation/extraction with acetone/H_2_O (80:20 *v/v*)/evaporation/liquid-liquid crude fractionation: solubilisation in H_2_O, extraction with chloroform/extraction with ethyl acetate	UHPLC-HRMS system equipped with an ESI-Q-TOF MS	[52,53]
Proanthocyanidin tannins (catechin, epicatechin for procyanidins Tannins; gallocatechin, epigallocatechin for prodelphinidins tannins)/grape seeds, skins and stems	Lyophilisation and extraction with ethanol/acidified H_2_O (1:1; *v*/*v*) under nitrogen, then with chloroform or extraction with acetone/H_2_O (7:3, *v/v*) and lyophilisation	Bate–Smith reaction (total content of proanthocyanidins)/thioacidolysis/HPLC-ESI-MS/MALDI-ToF-MS	[54]
Condensed tannins/*V. vinifera* skins	Freezing, skin separation, extraction with acetone/H_2_O (3:2 *v/v*), concentration, dissolving in ethanol/H_2_O/trifluoroacetic acid (11:9:0.001) for analysis, the acidolysis of extracts: hydrolysis with 5% solution of toluene-alpha-thiol in methanol containing 0.2 M hydrochloric acid, 60 °C, 10 min.	LC-MS, NMR	[55,56]
Stilbenoids/grape canes	Extraction in acetone/H_2_O mixture (6:4) overnight at room temperature, and the dry extract suspension in methanol/H_2_O (1:1)	LC-MS, (A) H_2_O 0.1% formic acid and (B) acetonitrile 0.1% formic acid NMR	[51]

**Table 3:** Possible applications of *V. vinifera* in cosmetic production according to the CosIng database.

INCI Name	Indications
*Vitis vinifera*	Skin protecting, fragrance
*Vitis vinifera* bud extract	Skin conditioning
*Vitis vinifera* callus culture-conditioned media	Antioxidant, skin conditioning
*Vitis vinifera* callus extract	Skin protecting
*Vitis vinifera* callus powder	Skin conditioning
*Vitis vinifera* flower cell extract	Fragrance, skin protecting
*Vitis vinifera* flower extract	Fragrance, skin conditioning, emollient
*Vitis vinifera* fruit cell extract	Skin conditioning
*Vitis vinifera* fruit extract	Skin conditioning
*Vitis vinifera* fruit juice ferment less oil	Fragrance, perfuming
*Vitis vinifera* fruit meristem cell culture	Antioxidant, skin protecting
*Vitis vinifera* fruit powder	Antioxidant, skin conditioning
*Vitis vinifera* fruit water	Skin conditioning
*Vitis vinifera* juice	Antioxidant, skin conditioning
*Vitis vinifera* juice extract	Antioxidant, skin conditioning
*Vitis vinifera* leaf cera	Skin protecting
*Vitis vinifera* leaf extract	Skin conditioning
*Vitis vinifera* leaf oil	Fragrance
*Vitis vinifera* leaf water	Skin conditioning
*Vitis vinifera* leaf wax	Skin protecting
*Vitis vinifera* leaf/seed/skin extract	Antioxidant
*Vitis vinifera* root extract	Skin conditioning
*Vitis vinifera* seed	Skin conditioning
*Vitis vinifera* seed extract	Anti-seborrheic, antimicrobial, antioxidant, oral care, skin protecting, UV absorber
*Vitis vinifera* seed oil	Skin conditioning-emollient
*Vitis vinifera* seed powder	Skin conditioning-emollient
*Vitis vinifera* seed/skin/stem extract	Antioxidant
*Vitis vinifera* shoot extract	Antioxidant, skin conditioning
*Vitis vinifera* skin extract	Antioxidant
*Vitis vinifera* skin powder	Antioxidant, skin conditioning
*Vitis vinifera* stem extract	Skin conditioning, skin protecting
*Vitis vinifera* vine extract	Skin conditioning
*Vitis vinifera* vine sap	Skin conditioning

**Table 4:** Examples of *V. vinifera*-derived ingredients currently used in cosmetic products.

Manufacturer, Country	Trade Name, Form	INCI Name	Function
Panier des Sens (France) www.panierdessens.com	Active firming cream	*Vitis vinifera (grape) seed oil, Vitis vinifera (grape) fruit extract, Vitis vinifera leaf extract*	Moisturises, has firming properties, reduces the appearance of orange skin
Panier des Sens (France) www.panierdessens.com	Exfoliating Soap	*Vitis vinifera (grape) seed oil, Vitis vinifera fruit extract, Vitis vinifera leaf extract, Vitis vinifera seed powder*	Cleanses, exfoliates, smoothes skin
Caudalie (France) www.caudalie.com	Toner, Grape Water	*Vitis vinifera (grape) fruit water, Vitis vinifera (grape) juice*	Smoothes, refreshes, moisturises, prevents redness
Apivita (Greece) www.apivita.com	Face Mask Line Reducing with Grape	*Vitis vinifera (Grape) seed oil*	Regenerates, moisturises, reduces wrinkles, soothes irritations
Korres (Greece) www.korres.com	Red Grape Sheer Glow Daily Sunscreen Face Cream	*Vitis vinifera fruit extract Korres Santorini grape fruit extract, Vitis vinifera grape fruit cell extract*	Protects against photoaging, reduces the visibility of discolourations and wrinkles
FarmStay (South Korea) en.fscos.com	Grape stem cell whitening lifting essence	*Vitis vinifera (grape) callus culture extract*	Inhibits the process of ageing, brightens, regenerates, firms, protects against UV
Organique (Poland) www.organique.pl	Peeling Intense Anti-ageing/Grape	*Vitis vinifera (Grape) Seed Oil, Grape Seed Powder (Vitis vinifera)*	Protects the skin from ageing, exfoliates, stimulates microcirculation, stimulates the penetration of active ingredients
Josh Rosebrook (The United States) joshrosebrook.com	Nourish Shampoo	*Grape Seed oil*	Gently cleanses, removes sebum excess, moisturises, softens hair, stimulates blood circulation
Caudalie (Paris) www.caudalie.com	Vinosource-Hydra, Grape Water Gel Moisturiser	*Vitis vinifera (Grape) Fruit Water, Vitis vinifera (Grape) Juice*	Moisturises, soothes irritations, strengthens the skin’s protective barrier
	Vinoperfect Instant Brightening Moisturiser	*Palmitoyl Grapevine Shoot Extract*	Moisturises, brightens, prevents discolourations
	Vinoperfect Glycolic Peel Mask	*Vitis vinifera (Grape) Seed Oil, Palmitoyl Grapevine Shoot Extract*	Gently exfoliates, brightens
	Resveratrol Lift Serum	*Grape vine Shoot Extract*	Moisturises, has firming properties, brightens
Die Nikolai (Austria) www.dienikolai.at	Grapeseed Intensive Serum	*Vitis vinifera (Grape) Seed Oil, Vitis vinifera (Grape) Seed Extract*	Eliminates free radicals, regenerates and repairs skin damage
Organique (Poland) www.organique.pl	Body Butter Anti-ageing/Grape	*Vitis vinifera Extract, Vitis vinifera (Grape) Seed Oil*	Moisturises, smoothes, nourishes, regenerates and slows down the ageing process

**Table 6:** Biological activity of *V. vinifera* for the potential applications for the treatment of dermatological problems.

Biological Activity	Tested Plant Material	Mechanism of Action	References
Antimicrobial	Pomace	Inhibition against: *B. cereus*, *S. aureus*, *C. albicans*, *C. krusei*	[63]
	Fruit juice	Inhibition against: *S. aureus*, *L. monocytogenes*, *S. epidermidis*, *E. hirae*, *S. pneumoniae*, *B. subtilis*, *S. pyogenes*, *E. durans*, *S. mutans*, *M. catarrhali*	[64]
	Fruit skin	*E. faecallis*, *S. aureus*, *E. aerogenes*	[65]
Anti-inflammatory	Dried fruit	-interleukin IL-8, (NF)-κB inhibition	[7]
	Seed	- albumin denaturation assay	[66]

## Data Availability

Not applicable.
